# Potential use of salivary TNF-α as a vaccine-induced pain biomarker in people with cerebral palsy and communication disorders

**DOI:** 10.1371/journal.pone.0308386

**Published:** 2024-12-27

**Authors:** Álvaro Sabater-Gárriz, José Joaquín Cerón, Pedro Montoya, Inmaculada Riquelme

**Affiliations:** 1 Balearic ASPACE Foundation, Marratxí, Spain; 2 Research Institute on Health Sciences (IUNICS-IdISBa), University of the Balearic Islands, Palma de Mallorca, Spain; 3 Department of Nursing and Physiotherapy, University of the Balearic Islands, Palma de Mallorca, Spain; 4 Interdisciplinary Laboratory of Clinical Analysis, Interlab-UMU, Regional Campus of International Excellence Campus Mare Nostrum, University of Murcia, Murcia, Spain; Universitas Indonesia Fakultas Kedokteran, INDONESIA

## Abstract

**Background:**

Pain in people with cerebral palsy (CP) has been classically underestimated and poorly treated, particularly in individuals with impaired communication skills.

**Objective:**

To analyze changes in different salivary metabolites and pain behavior scales after a painful procedure in adults with CP and adults with typical development.

**Methods:**

Salivary levels of sTNF-α, sIgA, Cortisol, FRAP, ADA and Alpha Amylase, as well as 3 observational pain scales (Wong-Baker, Non-Communicating Adults Pain Checklist and Facial Action Coding System) were assessed before and after an intramuscular injection in 30 Individuals with CP and 30 healthy controls. Video recording of face expression was performed during the procedure for offline analysis.

**Results:**

Pain in subjects with CP was higher than in healthy controls after the intramuscular injection as displayed by observational scales. sTNF-α experienced a significant post-stimulus increase in both groups and that increase shows a tendency to correlate with the observational scales scores. Other biomarkers classically associated with stress (cortisol, Alpha Amylase) remain stable.

**Conclusion:**

sTNF-α might be a promising pain indicator. Further research using controlled painful stimuli of greater intensity and pain self-reports, would be necessary to better understand its use as a pain biomarker.

## 1. Introduction

Pain in people with disabilities and communication disorders has been classically underestimated and poorly treated [1–4]. More than a third of children and 74% of young adults with cerebral palsy (CP) reports pain [5], usually as a consequence of motor deficiencies and the consequent effects on the structure and function of the organism that characterize CP [6–10]. Being identified as one of the main limitations to carry out activities of daily living [11] it has been argued that pain in people with CP may also occur because they are regularly exposed to painful health procedures [5, 12, 13].

Verbal communication is considered the "gold standard" for the assessment of pain [14, 15]. Thus, pain in populations with disabilities and communication deficits can be difficult to recognize by professionals [16], and caregivers [17, 18]. That, leads to a reduced or incomplete pain management [19]. It has been suggested that behavioral observational scales could help in evaluating pain in this collective [20]. However, there are other tools that have been validated for clinical practice and appears to be less dependent on observer’s subjective judgment. Thus, for example, the Facial Action Coding System (FACS) [21] allows categorizing the contraction of the muscles of facial expression for the detection of pain [22].

Furthermore, the use of biomarkers such as brain activity [23–25], skin conductance, muscle tension or heart rate variability [17, 26] could be used as objective indicators of pain to complement data obtained by observing behavior [27]. Determination of salivary metabolites could be a non-invasive, accurate and cost-effective method for the detection of pain in people with disabilities [28]. For example, cortisol [29], salivary Alpha Amylase (sAA), or secretory IgA (sIgA) [30, 31], adenosine deaminase (ADA) or Ferric Reducing Ability of Plasma (FRAP) [32–36] can be determined in saliva and are linked to the activation of the nociceptive/immune system [37]. Tumor Necrosis Factor-alpha (TNF-α) has also shown significant correlations with observational pain scales [27, 38] and disease severity [39]. TNF-α emerges as a potential biomarker for pain, influencing both peripheral and central sensitization, due to its implication in neuroinflammation and excitotoxicity [40]. In this sense, existing literature supports its role as a predictive biomarker of the transition from acute to chronic pain, in view of its implication in mechanisms of hyperalgesia and nociceptive plasticity [41–44]. Similarly, it can be used as a diagnostic marker, with associations noted in various acute and chronic painful conditions, such as neuropathic pain, myofascial pain and acute low back pain [45–47]. The behavior of TNF-α, showing an increment after acute pain in healthy populations [48], suggests its involvement in nociceptive processes, highlighting its potential for express pain produced by painful procedures.

The use of metabolites as diagnostic/prognostic biomarkers in CP has seen a surge in recent years, although the majority of the evidence focuses on the pediatric population. Several salivary cytokines, chemokines, hormones, and neuropeptides, such as agouti-related peptide, prolactin, cortisol, dynorphin, neuropeptide Y, somatostatin and nerve growth factor, have successfully differentiated pain from no-pain subgroups in children with CP [49]. In addition, salivary cortisol has been identified as a potential biomarker of stress and acute pain after rehabilitation programs [50]. In the adult population, different metabolic biomarkers such as such as cortisol, Insulin-like Growth Factor 1or serum creatinine have been successfully used to correlate with mental health and/or quality of life [51, 52] or other comorbidities such as cardiorespiratory morbidity and mortality, cardiovascular /cardiorespiratory disease risk factors, fractures, or metabolic syndrome [53, 54]. To the best of our knowledge, there is no evidence discussing the utility of metabolites in identifying the presence of acute pain in adults with CP. Therefore, it is our intention to contribute to filling this gap in the literature.

The present study explores the role of salivary metabolites sTNF-α, sIgA, sAA, cortisol, ADA and FRAP before and after a painful event (intramuscular injection) in adults with CP and healthy individuals. Pain perception will be evaluated using pain thresholds and three different observation scales to check the validity of these metabolites as pain biomarkers. We hypothesize that the mentioned markers, especially sTNF-α and cortisol, will experience an increase in their expression after the intramuscular injection, in both the group of individuals with CP and the control group.

## 2. Methods

### 2.1 Ethical considerations

Authorization for the participation of individuals with CP in all aspects of the protocol was obtained through their parents or legal representatives. In all cases, written informed consent was obtained. All the documents were written in standard form and in easy read format to allow its reading to as many participants with intellectual handicaps as possible. Healthy controls were informed as well and signed the informed consent. The study was performed in accordance with the Declaration of Helsinki (1991) and the protocol was approved by the Research Ethics Committee of [blinded for review] and by the Ethics Committee of [blinded for review].

### 2.2 Study design

This has been a cross-sectional study performed between Feb 2021 and Jan 2022.

### 2.3 Participants

The calculation of the sample size was carried out using the G*Power software [55] and taking TNF-alpha salival test as primary outcome, considering that the objective of the study was to detect changes in their expression before and after a painful stimulus. As the prevalence of cerebral palsy is estimated to be 1.5 cases/1000 inhabitants [56] (Bell et al, 2023) and the TNF alpha saliva test sensitivity is 10.7 pg/ml [57], accepting an alpha risk of 0.05 in a bilateral contrast, 30 subjects per group were needed to detect a difference equal to or greater than 10.7 (SD = 9) pg/ml in the TNF alpha saliva test.

A group of seventy-five adults (over 18 years of age) with CP and/or their legal representatives, users of the Adult Life Promotion Services of the Cerebral Palsy Association (ASPACE) of the Balearic Islands (Majorca, Spain) were contacted after the health staff verified that they did not meet the exclusion criteria. The contact was made by phone by the center’s management. The only exclusion criteria were being younger than 18 years and reporting recent surgery or presenting some type of acute inflammatory process in the last 6 months. Thirty people with CP (mean age = 40.30 (11.58) yr., age range = 21–69 yr.; 10 females) agreed to participate and their respective legal representatives signed the informed consent. In addition, A administrative officer from ASPACE Balearic Foundation contacted 110 professionals from the organization to invite them to participate in the study, of which 42 accepted. Finally, 30 (mean age = 38.87 (9.12) yr., age range = 25–64 yr.; 10 females) were selected with the priority of matching the gender and age representation of the CP group. S1 Table (see S1 Table) displays the Consort Flow chart to illustrate the selection, enrollment, and adherence of participants.

### 2.4 Measures

Sociodemographic and clinical variables (i.e. CP subtype, hand dominance, level of gross motor and communicative function) were reported or collected from the patient’s medical records. Moreover, the following measures were assessed:

#### 2.4.1 Salivary metabolites

Values of sTNF-α, sIgA, cortisol, FRAP, ADA and sAA were determined before and after an intramuscular injection. Total sIgA was quantified with a commercial ELISA kit (Bethyl, USA) previously validated for use with human saliva samples [58]. Cortisol was measured using a chemiluminescence assay (Siemens, USA) validated for use with human saliva samples [59]. The measurement of FRAP was based on the method described by Benzie and Strain with some modifications [60]. The sample was mixed without previous dilution with a reagent containing ferric-tripyridyltriazine complex which is reduced to ferrous-tripyridyltriazine by the non-enzymatic antioxidants of the sample. A first and second read of the reaction were taken just after the assay mixture and 240 s after, respectively, by using the Olympus AU400 automated biochemistry analyzer. A standard curve with different concentrations of ferrous ion was used to calculate the concentration of the antioxidant capacity of the samples. sAA activity was measured using a commercial colorimetric kit (Alpha-Amylase, Beckman Coulter Inc., Fullerton, CA, USA) following the IFCC method as previously reported [61]. ADA was measured using a previously validated spectrophotometric assay [62]. sTNF-α was measured using a commercially available ELISA kit (Diaclone, France) that was previously used on saliva samples [63]. Salivary biomarkers have been able to identify stress and pain in other studies with individuals with CP [50].

#### 2.4.2 Observational scales

**The Non-Communicating Adults Pain Checklist** (NCAPC) [64] classifies pain behaviors into six domains: vocal response, emotional response, facial expression, body language, protective responses, and physiological responses. It is based on the Non-Communicating Children Pain Checklist (NCCPC), a scale that proved to be the easiest to use without depending on familiarity with the subject or their level of development [65]. The NCAPC has shown good psychometric properties and the ability to differentiate not only between the presence and absence of pain, but also between different pain intensities in adults with intellectual and developmental disabilities [64]. As previously mentioned, recent evidence points to this scale as the one of election to assess pain this population.

**The Wong Baker FACES® Pain Rating Scale** [66] is a pain measurement tool rating pain from 0 (no pain) to 10 (the worst pain possible). Although it is used primarily in pediatric populations hospital, it is also a common scale for assessing pain in the field of disability and in patients with communication disorders [67].

The **Facial Action Coding System** (FACS) [21] is a widely used method for coding facial expressions in research [68] and it has been used previously in individuals with CP and communications disorders [24]. It was designed to provide objective descriptions of facial activity while reducing the possibility of subjective judgments. It is based on the identification and description of 44 facial action units (AU) by the examination of slow-motion videos. FACS classifies the frequency and intensity of each UA on a 6-point scale (0 = no expression, 5 = extreme expression). The expression of pain is coded in the following AU’s: lowering of the eyebrows (AU4), elevation of the cheeks and compression of the eyelids and/or contraction of the cheekbones (AU6/AU7), wrinkling of the nose (AU9), raising the upper lip (AU10) and closing the eyes (AU43) [69, 70]. The final score is obtained by the sum of AU4, AU6 or AU7 (whichever is higher), AU9 or AU10 (whichever is higher) and AU43 to make up a 20-point scale.


Pain=AU4+(AU6‖AU7)+(AU9‖AU10)+AU43

#### 2.4.3 Composite index

In order to deepen the understanding of how the different variables reflect a global response and inspired by the idea underlying the concept of allostatic load [71], a Composite Index of these biomarkers was calculated.

Allostatic load can be interpreted as the “‘wear and tear’ the body experiences when repeated allostatic responses are activated during stressful situations” [72]. Mediated by several hormones, neurotrophins, neurotransmitters, oxidative stress and immune-inflammatory response markers, allostatic load is a reflection of an adaptive response to some stressors overlaid by additional loads from unpredictable events in the environment, such as disease, human disturbances, and social interactions [73]. In those situations, the allostatic load can increase dramatically, being a useful measure for predicting unfavorable stress-related outcomes [74, 75]. To perform the calculation of the Composite Index, we grouped the variables into 4 categories: (1) Observational Markers (EVA, NCAPC, FACS), (2) Neuroendocrine Markers (Cortisol), (3) Immune Markers (sTNF-α, ADA, sIgA), and (4) Oxidative stress markers (sAA, FRAP). The 75th percentile of each marker was determined based on the distribution in the study sample. Subsequently, a "scaling" approach used previously for computing allostatic load [76, 77] was adapted for our calculation. Thus, for each individual, each marker with values above the 75th percentile was scored as ’1’ and the markers with values below the 75th percentile, as ‘0’. The sum of all markers in each category (observational, immune, neuroendocrine, and oxidative stress) was divided by the number of markers in each category in order to allow equal weighting of all four. Finally, the scores of the four categories were added and a final CI was calculated.


IC=[(EVA_DICO+NCAPC_DICO+FACS_DICO)/3]+[Cortisol_DICO]+[(sTNF_DICO+ADA_DICO+sIgA_DICO)/3]+[(sAA_DICO+FRAP_DICO)/2]

#### 2.4.4 Tactile and pain thresholds

**Tactile thresholds** were assessed in non-painful body locations (back of the dominant hand, between the second and third metacarpals) with Von Frey monofilaments (Somedic Sales AB, Hörby Sweden) [78]. These filaments consist of 17 nylon hairs with rounded tips and diameters ranging from 0.14 to 1.01 mm that stimulate fine touch receptors. The filament was pushed perpendicular to the point to be explored until it bends, making three touches to ensure that the response was coincident. After several practice trials, participants were asked to close their eyes/not look at the stimulated area and to notify if they felt any tactile sensation by saying “yes” or “no”. Null stimuli were applied randomly (once every 4–5 stimuli) to check for false positive responses. In this case, participants were informed that they had perceived a null stimulus in order to refocus their attention, but response was not included for threshold calculation. Responses with a delay of more than 3s were considered as undetected. Previous studies have shown the usefulness of this procedure to measure somatosensory sensitivity in adults with CP [79].

**Pressure pain thresholds** (expressed in N/cm2) were measured with a digital dynamometer (Force One, Wagner Instruments, Greenwich, CT USA) with a flat rubber tip (1 cm^2^) in the same body locations as described above. Participants were asked to say “pain” or to raise a hand when the pressure became painful. In those cases in which the participant could not express himself with words or gestures, the response to the painful stimulus was determined by observing the behavior by a professional with experience in the daily care of the participant, according to the consensus of Backonja et al. [80]. This procedure has been successfully used for determining pain sensitivity in adults with CP [79]. Two stimuli were applied and the pressure pain threshold was taken as the mean intensity at which the participants experienced an uncomfortable sensation.

### 2.5 Procedure

Saliva samples were collected before and 10-minutes after a painful procedure (intramuscular injection: BioNTech, Pfizer vaccine to protect against COVID-19 in participants with CP and physiological saline injection—0.9% sodium chloride—in healthy controls). All injections were administered in the deltoid muscle belly and were videotaped for off-line analysis of NCAPC and FACS, which was performed by two independent clinicians. During the intramuscular injection, each participant’s facial expression was scored by a trained clinician using the Wong Baker FACES®.

Professionals from the ASPACE Balearic Foundation received training on how to collect saliva samples using the SalivaBio Children’s Swab (SCS) kit from Salimetrics following the manufacturer’s instructions (https://salimetrics.com/wp-content/uploads/2018/02/children-swab-saliva-collection-instructions.pdf). One hour before sample collection, tactile sensitivity and pain threshold were assessed. At that moment, participants were instructed not to practice physical exercise, eat, drink other beverages than water, brush their teeth or consume caffeine until the sample collection. Five minutes before sample collection, to reduce saliva contamination with possible leftover food, the participants’ mouths were rinsed with clean water. Before starting the sample collection, participants were asked to swallow any saliva present in the mouth. Participants with CP unable to understand the order were prepared by care professionals, who removed residual saliva from their mouths with the help of absorbent sponges commonly used for oral hygiene.

Two saliva samples were collected, one before and one after the painful procedure (intramuscular injection). The first saliva sample was collected 10 minutes prior to administering the intramuscular injection, by keeping a swab in the participant’s mouth for 2 minutes. The subsequent collection was carried out at 10 minutes post-injection following the same protocol. These times were selected considering evidence that indicates the temporal ability of the different metabolites to show changes [81]. Immediately after saliva collection, the swab was inserted into a 10ml syringe, through which the collected saliva was extracted into a polypropylene centrifuge tube using the compression method. The samples were immediately refrigerated and frozen at −80°C until they were analyzed.

### 2.6 Statistical analysis

The statistical package SPSS (V.22, IBM, Armonk, NY, United States) was used for analyses. Descriptive statistics were used to characterize the sample and the different study variables in both groups. Analyses of variance for repetitive measures (ANOVA) were performed with GROUP (individuals with CP vs. healthy controls) as between-subjects factor and TIME (before the intramuscular injection vs. after the intramuscular injection) as within-subject factors. Greenhouse–Geisser correction was applied for the violation of sphericity assumptions; Bonferroni corrections were applied for post-hoc comparisons. In addition, bivariant correlations were computed between clinical variables, metabolite levels and observational scales.

## 3. Results

Groups were similar in sensitivity to touch and pain pressure stimuli. Table 1 displays the clinical characteristics of participants with CP.

**Table 1:** Clinical characteristics of participants with cerebral palsy.

	n
**GMFCS**	
Level I	7/30
Level II	2/30
Level III	1/30
Level IV	11/30
Level V	9/30
**CFCS**	
Level 0	1/30
Level I	3/30
Level II	5/30
Level III	8/30
Level IV	7/30
Level V	6/30
**CP subtype**	
Mixed tetraplegia	7/30
Mixed spastic tetraplegia	2/30
Spastic tetraplegia	1/30
Spastic tetraparesis	11/30
Spastic quadruplegia	9/30

*CP*: *cerebral palsy; GMCFC*: *Gross Motor Function Classification System* (Palisano et al., 1997 [82]), *CFCS*: *Communication Function Classification System* (Hidecker et al., 2011 [83]). These scales classify the person into 5 levels of function: lower scores indicating lower impairment of function.

### 3.1 Salivary metabolites

**Fig 1:**
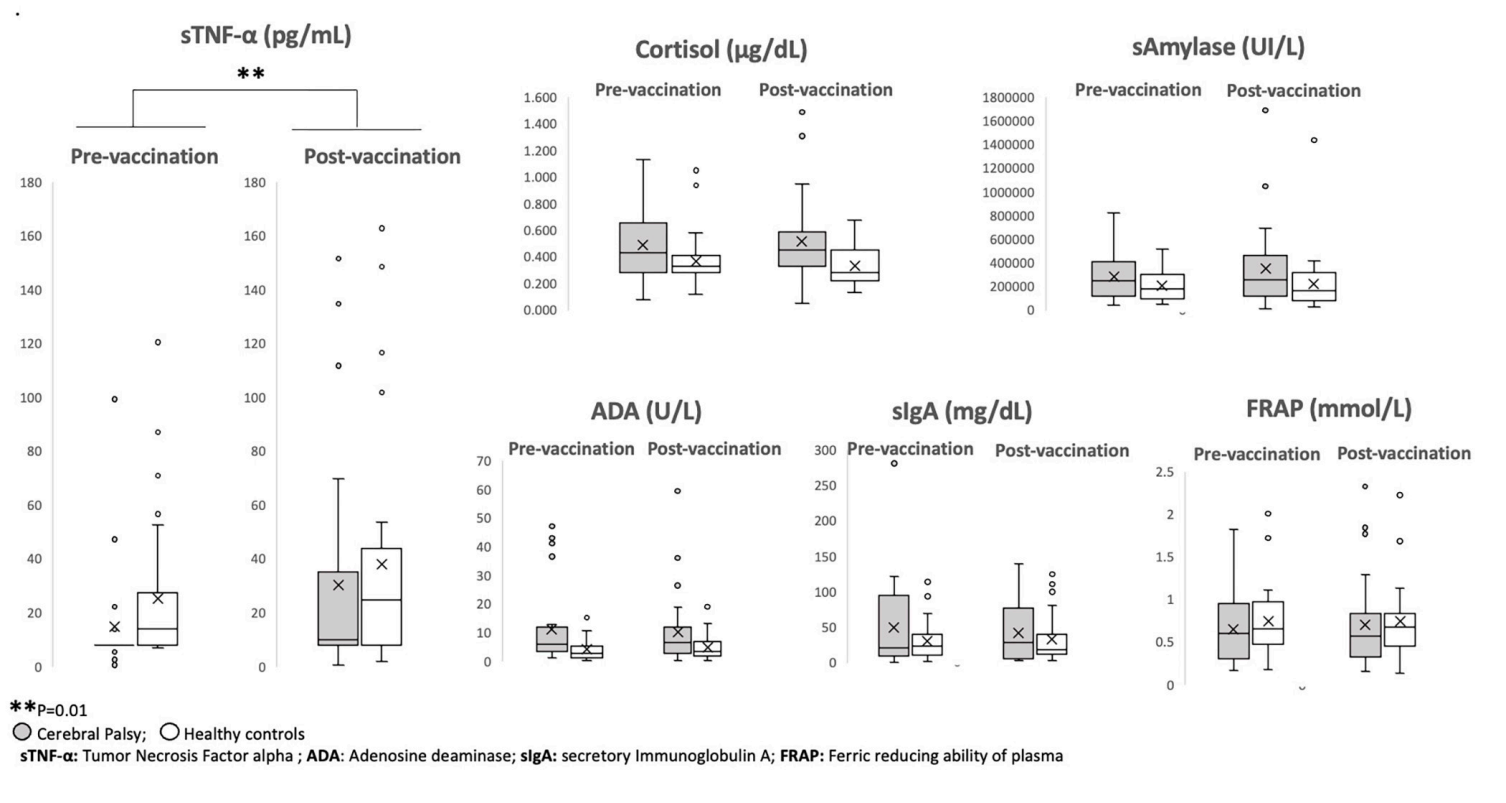
Box plot of the expression of selected salivary metabolites 10 min previous the intramuscular injection (pre-vaccination) and 10 minutes post injection (post-vaccination).

Fig 1 displays the distribution of salivary metabolites in both groups before and after the painful stimuli. sTNF-α showed a TIME effect (F[1,55] = 7.1, p = .010), showing that both groups had increased its expression after the intramuscular injection as compared with before the intramuscular injection. No GROUP nor TIME x GROUP effects were found. No significant statistical differences were found for the rest of the metabolites.

### 3.2 Pain observational scales

**Fig 2:**
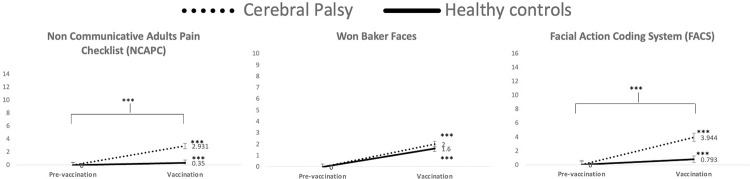
Graphical representation of the means and standard errors of the observational pain scales comparing 2 times: Before intramuscular injection (pre-vaccination) and at the time of injection (vaccination). ***Sig p<0.001.

Observational scales were scored by two independent evaluators. The Intraclass Correlation Coefficient (ICC) between the evaluators resulted of 0.65, 0.71 and 0.73 for Won-BakerFACES®, NCAPC and FACS respectively (reliability between fair and good). An average of the two scores was used for calculating the final score for the statistical analyses. Fig 2 displays means and typical errors of the observational scales of pain (Fig 2).

Pain scores using the Wong Baker FACES® showed a significant GROUP effect (F[1,58] = 11.1, p = .002), indicating more pain reported in individuals with CP than in healthy controls. In addition, a significant TIME effect was yielded (F[1,58] = 211.9, p < .001), showing that both groups had increased pain after the intramuscular injection as compared with before the intramuscular injection. However, there was no significant TIME x GROUP effect (F[1,58] = 2.6, p = .111).

The Non Communicative Adult Pain Checklist (NCAPC) indicated a significant GROUP effect (F[1,58] = 20.4, p = < .001), indicating that individuals with CP had more pain signs than healthy controls. In addition, a significant TIME effect was yielded (F[1,58] = 33.0, p < .001), showing that both groups had increased during the intramuscular injection as compared with before the intramuscular injection. There was a significant TIME x GROUP effect (F[1,58] = 20.4, p = < .001), showing higher pain expressions in individuals with PC than in the control group both before and during the intramuscular injection (both p < .001) and that only individuals with CP showed an increase of the painful behavior scores during the intramuscular injection (p < .001), whereas no change was observed in healthy controls (p = .803).

FACS scores indicated a significant GROUP effect (F[1,45] = 16.5, p = < .001), indicating that individuals with CP had more pain signs than healthy controls. In addition, a significant TIME effect was yielded (F[1,45] = 37.2, p < .001), showing that both groups had increased pain during the intramuscular injection as compared with before the intramuscular injection. There was a significant TIME x GROUP effect (F[1,45] = 16.5, p = < .001) showing higher pain expressions in individuals with PC than in the control group both before and during the intramuscular injection (both p < .001) and that only individuals with CP showed an increase of the painful behavior scores during the intramuscular injection (p < .001), whereas no change was observed in healthy controls (p = .106).

### 3.3 Composite index

**Fig 3:**
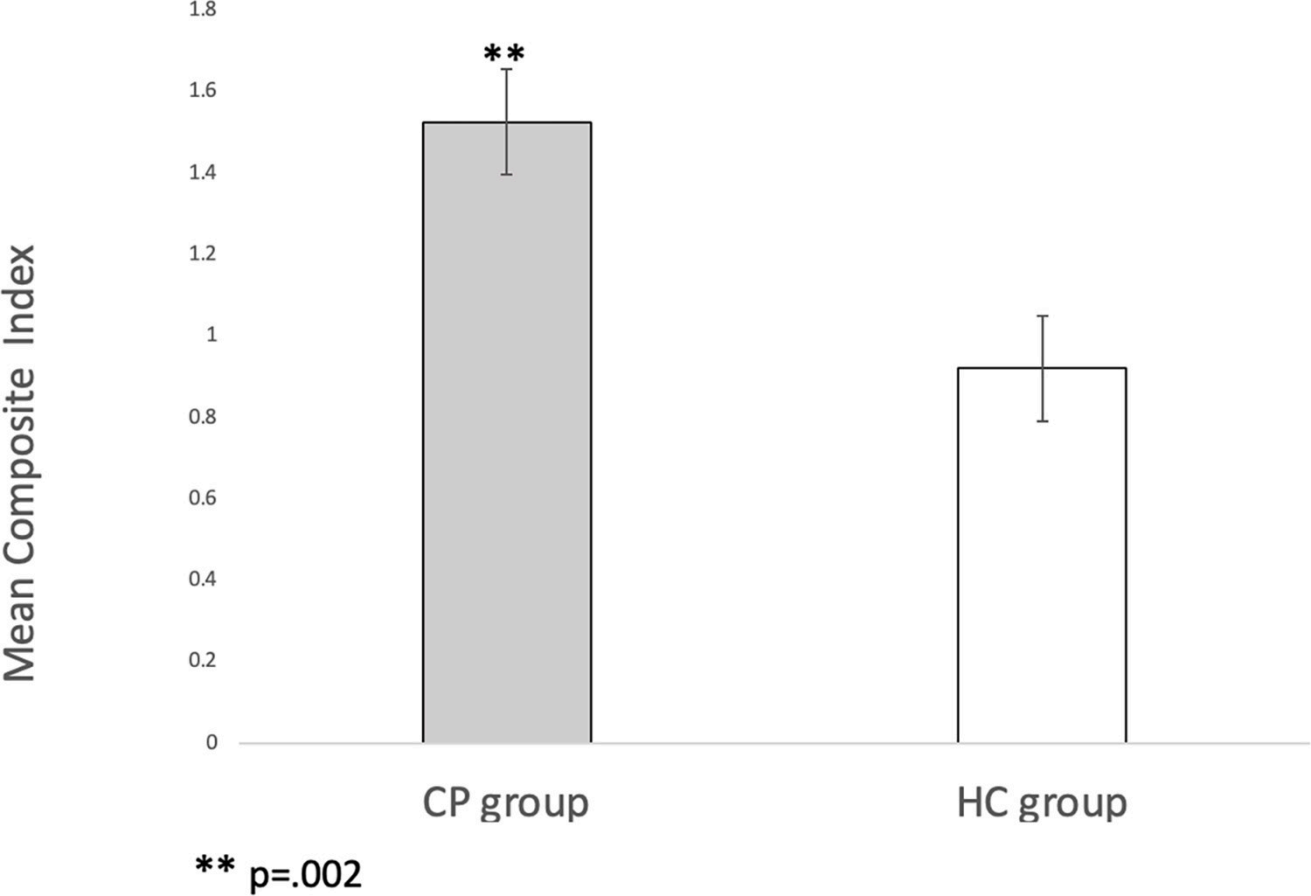
Means of the composite index after the vaccination.

The total composite index showed a significant GROUP effect (F[1,58] = 11.1, p = .002), indicating higher index in individuals with CP than in healthy controls (Fig 3). In addition, a significant TIME effect was yielded (F[1,58] = 211.9, p < .001), showing that both groups had increased the index after the vaccination as compared with before the vaccination. However, there was no significant TIME x GROUP effect (F[1,58] = 2.6, p = .111).

The analysis of the different categories of the composite index is displayed in Table 2. In general, all the categories showed an effect GROUP, revealing higher levels in individuals with CP than in healthy controls, and an effect TIME, expressing a significant increase in all the variables after the intramuscular injection except for the neuroendocrine category. Nevertheless, no interaction effects TIME x GROUP were found in any of the categories.

**Table 2:** Mean ± standard deviation of the different categories of the composite index before and after the vaccination.

	Before vaccination			After vaccination			TOTAL PRE-POST
	CP	HC		CP	HC		
**Observational**	0±0	0±0	p = 0	0.67±0,58	0.53±0,58	p = 0.11	**p = < .001**
**Neuroendocrine**	0.4±0.75	0.1±0.75	P = 0.007	0.33±0.73	0.1±0.73	P = 0.028	p = 0.644
**Immune**	0.28±0.49	0.22±0.49	p = 0.44	0.38±0.49	0.34±0.49	p = 0.52	**p = 0.002**
**Oxidative stress**	0.18±0.59	0.07±0.59	p = 0.04	0.28±0.59	0,17±0.59	p = 0.16	**p = 0.014**

Table 2 References: CP–Cerebral Palsy Group; HC–Healthy Controls

### 3.4 Tactile and pain thresholds

Baseline tactile thresholds were significantly higher in adults with CP than in control individuals (F(1,58) = 11.13, p = .001), indicating lower tactile sensitivity in individuals with CP. No differences between the groups were found in baseline pain thresholds (p = .877).

### 3.5 Correlations between salivary metabolites and observational pain scales

To perform the correlations, we calculated the percentage change of the values after the intramuscular injection with respect to before the intramuscular injection for all the variables under study.

The change in cortisol correlated with the change in NCAPC (r =.-433, p = .057 and r = .305, p = .033 respectively), revealing that an increase in pain perception during the intramuscular injection was mirrored by an increase of cortisol values after the intramuscular injection. In order to further explore these significant correlations, we analyzed both groups separately. Cortisol levels after the intramuscular injection correlate with change in NCAPC (r = -.396, p = .045) in individuals with CP, but not in healthy controls, indicating that higher cortisol levels were associated with less painful behaviors only in individuals with CP. No other correlations were found between metabolite levels and observational scales. Touch thresholds correlated with the change in the NCAPC in individuals with CP, but not in healthy individuals (r = .530, p = .004), revealing an association between lower baseline levels of tactile sensitivity (higher thresholds) with higher changes in painful behaviors in individuals with CP. No significant correlations were found between clinical variables and observational scores or metabolite changes.

## 4. Discussion

In order to explore the role of salivary metabolites as biomarkers of pain in people with CP and communication impairment, the aims of the present study were: 1) to analyze possible differences in pain perception between people with CP and HC when faced an acute painful stimulus, such as an intramuscular injection (vaccine), and 2) to explore the correlation of observational scales and baseline pain and tactile thresholds with the change in the expression of salivary metabolites. Although individuals with CP showed higher pain behaviors than healthy individuals, both groups had similar changes in the expression of salivary metabolites or a composite index joining behavioral and endocrine markers after an acute pain procedure.

Observational scales seem to be still the best tools for pain detection in individuals with CP, being able to detect changes in behaviors even in a stimulus classified as “mild”, as it was an intramuscular injection [84]. In that sense, individuals with CP have shown higher scores in the observational tools than HC, in concordance with previous research [14, 24, 68]. However, observational scales have shown limitations in the assessment of pain intensity in people unable to verbalize their pain in specific situations of spasticity or chronic pain [14, 24, 68]. Furthermore, a possible handicap of observational scales is that they often do not make clear what part of the observed responses is caused by pain or by other factors, such as fear or stress [85]. An in-depth pain assessment with other methods would be a good complement for detecting pain situations in individuals with CP.

Regarding salivary metabolites, sTNF-α showed increased values after painful stimulation for both individuals with CP and healthy controls. In contrast to other metabolites, such as cortisol or sAA, that have been clearly related to stress in people with typical development [35, 86] and individuals with CP [87], sTNF-α has been associated to pain perception in populations with typical development and elderly with dementia [27, 38]. In addition, it has been suggested that sTNF-α could be a biomarker of pain in patients with fibromyalgia [88] and that sTNF-α levels are reduced after anti-inflammatory treatments [39]. Therefore, although the correlation observed in this study between sTNF-α and the perception of pain measured with the observational scales is absent, it could be interpreted that the increase in sTNF-α, but not in the rest of the metabolites associated with stress, could be considered as a biomarker of pain also in individuals with CP. Thus, it should be noted that the fact that sTNF-α levels increased while cortisol levels remained stable could indicate that the procedure used (intramuscular injection), although slightly painful, was not stressful for individuals with CP or for the group of healthy individuals. However, the fact that behavioral pain scores were associated with cortisol levels in individuals with CP also suggests that this metabolite could be used as a biomarker of pain in this population. In this sense, previous studies have considered that the increase in cortisol levels could be understood as an indicator of stress, but also of pain in PC [50] and other populations, such as fibromyalgia and patients with rheumatoid arthritis [89, 90]. Further research should deepen in the role of cortisol in specific populations such as those with developmental disorders.

### 4.1 Study limitations

The main limitation of this study was due to the fact that the painful stimulus was of short duration and low intensity. This could have led to a low response on both the metabolite expression and observation scales. It would be convenient to examine whether stimuli of longer duration and intensity cause significant changes in the variables examined in this study. Although the acute pain procedure using a needle prick was similar in intensity and duration comparable in the participants with CP and the control group, the use of different solutions might have affected pain perception and bias the results. Although this procedure was included in the health routine of the cerebral palsy participants, minimizing additional pain discomfort, other procedures surpassing these limitations should be explored in future studies. The measures used in the study, although supported by previous research in individuals with cerebral palsy or similar conditions, were not specifically validated for this population; although the saliva sampling was chosen for being a quick, easy and low invasive method, supported by previous research [49, 50], and having the potential to be used in daily pain exploration, a blood sample might have proportionated more reliable information about pain-related metabolite changes. Furthermore, the use of a vaccine as a pain trigger may have influenced the levels of sTNF-α compared to other markers such as cortisol. Finally, due to the cognitive disability that many of the participants had, it was not possible to compare the scores obtained on the observational scales with self-reported pain to assess the agreement between them. In this sense, previous studies have revealed that the agreement between self-reports and the observation of pain by family members and healthcare personnel is relatively moderate in people with CP [13].

## 5. Conclusions

In conclusion, despite their subjective component, observational scales appear still as the most useful instruments for detecting the presence of pain in adults with CP with communication impairments. Although sTNF-α might be a promising pain indicator, further research using controlled painful stimuli of greater intensity and being able to compare observational measures with self-reported pain, are necessary to better understand its use as a pain biomarker. It would also be interesting to focus future research on the temporal behavior of the proposed biomarkers in order to further clarify their utility in diagnosing pain in adults with CP. Finding a more objective way for this diagnosis is crucial to improve the health and quality of life of this population, especially when they face communication impairments.

## Supporting information

S1 Checklist STROBE statement. (DOCX)

S1 Table Consort flow chart. (DOCX)

## References

[1] Axmon A, Sandberg M, Ahlström G, Midlöv P. Prescription of potentially inappropriate medications among older people with intellectual disability: a register study. BMC Pharmacol Toxicol. 2017 Oct 25;18(1):68. doi: 10.1186/s40360-017-0174-1 ; PMCID: PMC5657112. 29070067 PMC5657112

[2] Duerden EG, Taylor MJ, Lee M, McGrath PA, Davis KD, Roberts SW. Decreased sensitivity to thermal stimuli in adolescents with autism spectrum disorder: relation to symptomatology and cognitive ability. J Pain. 2015 May;16(5):463–71. doi: 10.1016/j.jpain.2015.02.001 Epub 2015 Feb 19. . 25704841

[3] McGuire BE, Daly P, Smyth F. Chronic pain in people with an intellectual disability: under-recognised and under-treated? J Intellect Disabil Res. 2010 Mar;54(3):240–5. doi: 10.1111/j.1365-2788.2010.01254.x . 20387264

[4] Rodby-Bousquet E, Alriksson-Schmidt A, Jarl J. Prevalence of pain and interference with daily activities and sleep in adults with cerebral palsy. Dev Med Child Neurol. 2021 Jan;63(1):60–67. doi: 10.1111/dmcn.14678 Epub 2020 Sep 19. ; PMCID: PMC7756851. 32951227 PMC7756851

[5] Mckinnon CT, Meehan EM, Harvey AR, Antolovich GC, Morgan PE. Prevalence and characteristics of pain in children and young adults with cerebral palsy: a systematic review. Dev Med Child Neurol. 2019 Mar;61(3):305–314. doi: 10.1111/dmcn.14111 Epub 2018 Dec 3. . 30508221

[6] Flanigan M, Gaebler-Spira D, Kocherginsky M, Garrett A, Marciniak C. Spasticity and pain in adults with cerebral palsy. Dev Med Child Neurol. 2020 Mar;62(3):379–385. doi: 10.1111/dmcn.14368 Epub 2019 Oct 10. . 31602643

[7] Holmes C, Brock K, Morgan P. Pain and its relationship with postural asymmetry in adults with cerebral palsy: A preliminary exploratory study. Disabil Health J. 2021 Jul;14(3):101063. doi: 10.1016/j.dhjo.2021.101063 Epub 2021 Jan 19. . 33509734

[8] Kinne S, Patrick DL, Doyle DL. Prevalence of secondary conditions among people with disabilities. Am J Public Health. 2004 Mar;94(3):443–5. doi: 10.2105/ajph.94.3.443 ; PMCID: PMC1448273. 14998811 PMC1448273

[9] McKearnan KA, Kieckhefer GM, Engel JM, Jensen MP, Labyak S. Pain in children with cerebral palsy: a review. J Neurosci Nurs. 2004 Oct;36(5):252–9. doi: 10.1097/01376517-200410000-00004 . 15524243

[10] Vogtle LK. Pain in adults with cerebral palsy: impact and solutions. Dev Med Child Neurol. 2009 Oct;51 Suppl 4:113–21. doi: 10.1111/j.1469-8749.2009.03423.x . 19740218

[11] du Toit J, Eken MM, Lamberts RP, Langerak NG. Adults with spastic diplegic cerebral palsy living in a low-to-middle income country: A six-year follow-up study on pain, functional mobility, activity and participation. Disabil Health J. 2021 Oct;14(4):101130. doi: 10.1016/j.dhjo.2021.101130 Epub 2021 Jun 8. . 34172416

[12] Ostojic K, Paget SP, Morrow AM. Management of pain in children and adolescents with cerebral palsy: a systematic review. Dev Med Child Neurol. 2019 Mar;61(3):315–321. doi: 10.1111/dmcn.14088 Epub 2018 Oct 31. . 30378122

[13] Riquelme I, Cifre I, Montoya P. Are physiotherapists reliable proxies for the recognition of pain in individuals with cerebral palsy? A cross sectional study. Disabil Health J. 2015 Apr;8(2):264–70. doi: 10.1016/j.dhjo.2014.08.009 Epub 2014 Aug 23. . 25258089

[14] Fox MA, Ayyangar R, Parten R, Haapala HJ, Schilling SG, Kalpakjian CZ. Self-report of pain in young people and adults with spastic cerebral palsy: interrater reliability of the revised Face, Legs, Activity, Cry, and Consolability (r-FLACC) scale ratings. Dev Med Child Neurol. 2019 Jan;61(1):69–74. doi: 10.1111/dmcn.13980 Epub 2018 Jul 27. . 30051908

[15] Raja SN, Carr DB, Cohen M, Finnerup NB, Flor H, Gibson S, et al. The revised International Association for the Study of Pain definition of pain: concepts, challenges, and compromises. Pain. 2020 Sep 1;161(9):1976–1982. doi: 10.1097/j.pain.0000000000001939 ; PMCID: PMC7680716. 32694387 PMC7680716

[16] McKinnon C, White J, Morgan P, Harvey A, Clancy C, Fahey M, et al. Clinician Perspectives of Chronic Pain Management in Children and Adolescents with Cerebral Palsy and Dyskinesia. Phys Occup Ther Pediatr. 2021;41(3):244–258. doi: 10.1080/01942638.2020.1847236 Epub 2020 Nov 29. . 33251932

[17] Bradley MM, Silakowski T, Lang PJ. Fear of pain and defensive activation. Pain. 2008 Jul;137(1):156–163. doi: 10.1016/j.pain.2007.08.027 Epub 2007 Sep 27. ; PMCID: PMC2519040. 17904289 PMC2519040

[18] McKinnon C., White J., Harvey A., Antolovich G., & Morgan P. (2022). Caregiver perspectives of managing chronic pain in children and adolescents with dyskinetic and mixed dyskinetic/spastic CP with communication limitations. Journal of pediatric rehabilitation medicine, 15(1), 69–81. doi: 10.3233/PRM-200770 34151872

[19] New perspectives on the definition of pain. Pain. 1996 Sep;67(1):3–6. doi: 10.1016/0304-3959(96)03135-1 . 8895225

[20] Huda Huijer Abu-Saad. Challenge of pain in the cognitively impaired. Lancet. 2000 Dec 2;356(9245):1867–8. doi: 10.1016/S0140-6736(00)03253-0 . 11130378

[21] Ekman P, Friesen WV. Investigator’s guide to the Facial Action Coding System. Palo Alto, CA: Consulting Psychologist Press; 1978.

[22] Lautenbacher S, Hassan T, Seuss D, Loy FW, Garbas JU, Schmid U, et al. Automatic Coding of Facial Expressions of Pain: Are We There Yet? Pain Res Manag. 2022 Jan 11;2022:6635496. doi: 10.1155/2022/6635496 ; PMCID: PMC8767386. 35069957 PMC8767386

[23] Arbour C, Gélinas C, Loiselle CG, Bourgault P. An exploratory study of the bilateral bispectral index for pain detection in traumatic-brain-injured patients with altered level of consciousness. J Neurosci Nurs. 2015 Jun;47(3):166–77. doi: 10.1097/JNN.0000000000000137 . 25943998

[24] Benromano T, Pick CG, Granovsky Y, Defrin R. Increased Evoked Potentials and Behavioral Indices in Response to Pain Among Individuals with Intellectual Disability. Pain Med. 2017 Sep 1;18(9):1715–1730. doi: 10.1093/pm/pnw349 . 28339959

[25] Benromano T, Pick CG, Merick J, Defrin R. Physiological and Behavioral Responses to Calibrated Noxious Stimuli Among Individuals with Cerebral Palsy and Intellectual Disability. Pain Med. 2017 Mar 1;18(3):441–453. doi: 10.1093/pm/pnw155 . 27473634

[26] Koenig J, Jarczok MN, Ellis RJ, Hillecke TK, Thayer JF. Heart rate variability and experimentally induced pain in healthy adults: a systematic review. Eur J Pain. 2014 Mar;18(3):301–14. doi: 10.1002/j.1532-2149.2013.00379.x Epub 2013 Aug 6. . 23922336

[27] Cantón-Habas V, Rich-Ruiz M, Moreno-Casbas MT, Ramírez-Expósito MJ, Martínez-Martos JM, Carrera-González MDP. Correlation between Biomarkers of Pain in Saliva and PAINAD Scale in Elderly People with Cognitive Impairment and Inability to Communicate. J Clin Med. 2021 Apr 1;10(7):1424. doi: 10.3390/jcm10071424 ; PMCID: PMC8037327. 33915996 PMC8037327

[28] Hu S, Loo JA, Wong DT. Human saliva proteome analysis and disease biomarker discovery. Expert Rev Proteomics. 2007 Aug;4(4):531–8. doi: 10.1586/14789450.4.4.531 . 17705710

[29] Alresayes S, Al-Aali K, Javed F, Alghamdi O, Mokeem SA, Vohra F, et al. Assessment of self-rated pain perception and whole salivary cortisol levels among adolescents with and without temporomandibular disorders. Cranio. 2021 Mar 25:1–7. doi: 10.1080/08869634.2021.1899697 Epub ahead of print. . 33764284

[30] Canigur Bavbek N, Bozkaya E, Isler SC, Elbeg S, Uraz A, Yuksel S. Assessment of salivary stress and pain biomarkers and their relation to self-reported pain intensity during orthodontic tooth movement: a longitudinal and prospective study. J Orofac Orthop. 2022 Sep;83(5):339–352. English. doi: 10.1007/s00056-021-00311-4 Epub 2021 Jun 25. . 34170330

[31] Christidis N, Baghernejad P, Deyhim A, Jasim H. Salivary Alpha-Amylase in Experimentally-Induced Muscle Pain. Diagnostics (Basel). 2020 Sep 20;10(9):722. doi: 10.3390/diagnostics10090722 ; PMCID: PMC7554812. 32962201 PMC7554812

[32] Briguglio G, Teodoro M, Italia S, Verduci F, Pollicino M, Coco M, et al. Salivary Biomarkers and Work-Related Stress in Night Shift Workers. Int J Environ Res Public Health. 2021 Mar 19;18(6):3184. doi: 10.3390/ijerph18063184 ; PMCID: PMC8003447. 33808679 PMC8003447

[33] Hendrix J, Nijs J, Ickmans K, Godderis L, Ghosh M, Polli A. The Interplay between Oxidative Stress, Exercise, and Pain in Health and Disease: Potential Role of Autonomic Regulation and Epigenetic Mechanisms. Antioxidants (Basel). 2020 Nov 23;9(11):1166. doi: 10.3390/antiox9111166 ; PMCID: PMC7700330. 33238564 PMC7700330

[34] Obayashi K. Salivary mental stress proteins. Clin Chim Acta. 2013 Oct 21;425:196–201. doi: 10.1016/j.cca.2013.07.028 Epub 2013 Aug 9. . 23939251

[35] Pulopulos MM, Baeken C, De Raedt R. Cortisol response to stress: The role of expectancy and anticipatory stress regulation. Horm Behav. 2020 Jan;117:104587. doi: 10.1016/j.yhbeh.2019.104587 Epub 2019 Oct 25. . 31639385

[36] Reuter S, Gupta SC, Chaturvedi MM, Aggarwal BB. Oxidative stress, inflammation, and cancer: how are they linked? Free Radic Biol Med. 2010 Dec 1;49(11):1603–16. doi: 10.1016/j.freeradbiomed.2010.09.006 Epub 2010 Sep 16. ; PMCID: PMC2990475. 20840865 PMC2990475

[37] Baral P, Udit S, Chiu IM. Pain and immunity: implications for host defence. Nat Rev Immunol. 2019 Jul;19(7):433–447. doi: 10.1038/s41577-019-0147-2 ; PMCID: PMC6700742. 30874629 PMC6700742

[38] Sobas EM, Vázquez A, Videla S, Reinoso R, Fernández I, Garcia-Vazquez C, et al. Evaluation of Potential Pain Biomarkers in Saliva and Pain Perception After Corneal Advanced Surface Ablation Surgery. Clin Ophthalmol. 2020 Mar 3;14:613–623. doi: 10.2147/OPTH.S225603 ; PMCID: PMC7060776. 32184550 PMC7060776

[39] Yoshida RA, Gorjão R, Mayer MPA, Corazza PFL, Guare RO, Ferreira ACFM, et al. Inflammatory markers in the saliva of cerebral palsy individuals with gingivitis after periodontal treatment. Braz Oral Res. 2019 Jul 1;33:e033. doi: 10.1590/1807-3107bor-2019.vol33.0033 . 31269113

[40] Olmos G, Lladó J. Tumor necrosis factor alpha: a link between neuroinflammation and excitotoxicity. Mediators Inflamm. 2014;2014:861231. doi: 10.1155/2014/861231 Epub 2014 May 21. ; PMCID: PMC4055424. 24966471 PMC4055424

[41] Reichling DB, Levine JD. Critical role of nociceptor plasticity in chronic pain. Trends Neurosci. 2009 Dec;32(12):611–8. doi: 10.1016/j.tins.2009.07.007 Epub 2009 Sep 24. ; PMCID: PMC2787756. 19781793 PMC2787756

[42] Sun B., Zhang H., Dong Y., Zhao L., Han J., & Liu M. Evaluation of the combination mode and features of p38 MAPK inhibitors: construction of different pharmacophore models and molecular docking. Molecular Simulation, (2019). 45, 975–984. doi: 10.1080/08927022.2019.1606426

[43] Sorkin L, Svensson CI, Jones-Cordero TL, Hefferan MP, Campana WM. Spinal p38 mitogen-activated protein kinase mediates allodynia induced by first-degree burn in the rat. J Neurosci Res. 2009 Mar;87(4):948–55. doi: 10.1002/jnr.21905 ; PMCID: PMC2644733. 18855936 PMC2644733

[44] Watkins LR, Goehler LE, Relton J, Brewer MT, Maier SF. Mechanisms of tumor necrosis factor-alpha (TNF-alpha) hyperalgesia. Brain Res. 1995 Sep 18;692(1–2):244–50. doi: 10.1016/0006-8993(95)00715-3 . 8548310

[45] Leung L, Cahill CM. TNF-alpha and neuropathic pain—a review. J Neuroinflammation. 2010 Apr 16;7:27. doi: 10.1186/1742-2094-7-27 ; PMCID: PMC2861665. 20398373 PMC2861665

[46] Grosman-Rimon L, Parkinson W, Upadhye S, Clarke H, Katz J, Flannery J, et al. Circulating biomarkers in acute myofascial pain: A case-control study. Medicine (Baltimore). 2016 Sep;95(37):e4650. doi: 10.1097/MD.0000000000004650 ; PMCID: PMC5402557. 27631214 PMC5402557

[47] Klyne DM, Barbe MF, van den Hoorn W, Hodges PW. ISSLS PRIZE IN CLINICAL SCIENCE 2018: longitudinal analysis of inflammatory, psychological, and sleep-related factors following an acute low back pain episode-the good, the bad, and the ugly. Eur Spine J. 2018 Apr;27(4):763–777. doi: 10.1007/s00586-018-5490-7 Epub 2018 Feb 19. . 29460011

[48] Sobas EM, Reinoso R, Cuadrado-Asensio R, Fernández I, Maldonado MJ, Pastor JC. Reliability of Potential Pain Biomarkers in the Saliva of Healthy Subjects: Inter-Individual Differences and Intersession Variability. PLoS One. 2016 Dec 1;11(12):e0166976. doi: 10.1371/journal.pone.0166976 ; PMCID: PMC5132231. 27907037 PMC5132231

[49] Symons FJ, ElGhazi I, Reilly BG, Barney CC, Hanson L, Panoskaltsis-Mortari A, et al. Can biomarkers differentiate pain and no pain subgroups of nonverbal children with cerebral palsy? A preliminary investigation based on noninvasive saliva sampling. Pain Med. 2015 Feb;16(2):249–56. doi: 10.1111/pme.12545 Epub 2014 Sep 19. ; PMCID: PMC4332832. 25234580 PMC4332832

[50] Zhao X, Chen M, Du S, Li H, Li X. Evaluation of stress and pain in young children with cerebral palsy during early developmental intervention programs: a descriptive study. Am J Phys Med Rehabil. 2015 Mar;94(3):169–75; quiz 176–9. doi: 10.1097/PHM.0000000000000252 . 25500686

[51] Gorter JW, Fehlings D, Ferro MA, Gonzalez A, Green AD, Hopmans SN, et al. Correlates of Mental Health in Adolescents and Young Adults with Cerebral Palsy: A Cross-Sectional Analysis of the MyStory Project. J Clin Med. 2022 May 29;11(11):3060. doi: 10.3390/jcm11113060 ; PMCID: PMC9181041. 35683448 PMC9181041

[52] Ng TKS, Heyn PC, Tagawa A, Coughlan C, Carollo JJ. Associations of Circulating Insulin-Growth Factor-1 With Cognitive Functions and Quality of Life Domains in Ambulatory Young Adults With Cerebral Palsy: A Pilot Study. Front Neurol. 2022 Jun 27;13:748015. doi: 10.3389/fneur.2022.748015 ; PMCID: PMC9271561. 35832183 PMC9271561

[53] Heyn PC, Tagawa A, Pan Z, Thomas S, Carollo JJ. Prevalence of metabolic syndrome and cardiovascular disease risk factors in adults with cerebral palsy. Dev Med Child Neurol. 2019 Apr;61(4):477–483. doi: 10.1111/dmcn.14148 Epub 2019 Jan 20. . 30663044

[54] Whitney DG, Hurvitz EA. The age-related association between serum creatinine and cardiorespiratory morbidity and mortality and fractures among adults with cerebral palsy. Adv Med Sci. 2023 Jul 18;68(2):249–257. doi: 10.1016/j.advms.2023.07.001 Epub ahead of print. . 37473639

[55] Faul F., Erdfelder E., Lang A. G., & Buchner A. G* Power 3: A flexible statistical power analysis program for the social, behavioral, and biomedical sciences. Behavior research methods (2007) 39(2), 175–191. doi: 10.3758/bf03193146 17695343

[56] Bell BG, Shah S, Coulson N, McLaughlin J, Logan P, Luke R, et al. The impact of ageing on adults with cerebral palsy: the results of a national online survey. BJGP Open. 2023 Dec 12:BJGPO.2023.0028. doi: 10.3399/BJGPO.2023.0028 Epub ahead of print. . 37591553 PMC11176684

[57] https://www.diaclone.com/product/860.040.048-murine-tnf-%25CE%25B1-elisa-kit/732

[58] Lopez-Jornet P, Cayuela CA, Tvarijonaviciute A, Parra-Perez F, Escribano D, Ceron J. Oral lichen planus: salival biomarkers cortisol, immunoglobulin A, adiponectin. J Oral Pathol Med. 2016 Mar;45(3):211–7. doi: 10.1111/jop.12345 Epub 2015 Jul 27. . 26216173

[59] Tecles F, Fuentes-Rubio M, Tvarijonaviciute A, Martínez-Subiela S, Fatjó J, Cerón JJ. Assessment of stress associated with an oral public speech in veterinary students by salivary biomarkers. J Vet Med Educ. 2014 Spring;41(1):37–43. doi: 10.3138/jvme.0513-073R1 . 24449705

[60] Tvarijonaviciute A, Aznar-Cayuela C, Rubio CP, Ceron JJ, López-Jornet P. Evaluation of salivary oxidate stress biomarkers, nitric oxide and C-reactive protein in patients with oral lichen planus and burning mouth syndrome. J Oral Pathol Med. 2017 May;46(5):387–392. doi: 10.1111/jop.12522 Epub 2016 Dec 11. . 27862315

[61] Contreras-Aguilar MD, Escribano D, Martínez-Subiela S, Martínez-Miró S, Rubio M, Tvarijonaviciute A, et al. Influence of the way of reporting alpha-Amylase values in saliva in different naturalistic situations: A pilot study. PLoS One. 2017 Jun 27;12(6):e0180100. doi: 10.1371/journal.pone.0180100 ; PMCID: PMC5487069. 28654668 PMC5487069

[62] Franco-Martínez L, Tecles F, Torres-Cantero A, Bernal E, San Lázaro I, Alcaraz MJ, et al. Analytical validation of an automated assay for the measurement of adenosine deaminase (ADA) and its isoenzymes in saliva and a pilot evaluation of their changes in patients with SARS-CoV-2 infection. Clin Chem Lab Med. 2021 Apr 28;59(9):1592–1599. doi: 10.1515/cclm-2021-0324 . 33908223

[63] Polz-Dacewicz M, Strycharz-Dudziak M, Dworzański J, Stec A, Kocot J. Salivary and serum IL-10, TNF-α, TGF-β, VEGF levels in oropharyngeal squamous cell carcinoma and correlation with HPV and EBV infections. Infect Agent Cancer. 2016 Aug 20;11:45. doi: 10.1186/s13027-016-0093-6 ; PMCID: PMC4992298. 27547238 PMC4992298

[64] Lotan M, Moe-Nilssen R, Ljunggren AE, Strand LI. Measurement properties of the Non-Communicating Adult Pain Checklist (NCAPC): a pain scale for adults with Intellectual and Developmental Disabilities, scored in a clinical setting. Res Dev Disabil. 2010 Mar-Apr;31(2):367–75. doi: 10.1016/j.ridd.2009.10.008 Epub 2009 Nov 8. . 19900787

[65] Breau LM, Camfield CS. The relation between children’s pain behaviour and developmental characteristics: a cross-sectional study. Dev Med Child Neurol. 2011 Feb;53(2):e1–7. doi: 10.1111/j.1469-8749.2010.03842.x Epub 2010 Dec 1. . 21121907

[66] Wong DL, Baker CM. Pain in children: comparison of assessment scales. Pediatr Nurs. 1988 Jan-Feb;14(1):9–17. . 3344163

[67] Ozkan D, Gonen E, Akkaya T, Bakir M. Popliteal block for lower limb surgery in children with cerebral palsy: effect on sevoflurane consumption and postoperative pain (a randomized, double-blinded, controlled trial). J Anesth. 2017 Jun;31(3):358–364. doi: 10.1007/s00540-017-2318-2 Epub 2017 Feb 14. . 28197774

[68] Bartlett M., Littlewort G., Vural E., Lee K., Cetin M., Ercil A., et al. Data mining spontaneous facial behavior with automatic expression coding. Verbal and Nonverbal Features of Human-Human and Human-Machine Interaction).2008. (pp.1–20 Springer, Berlin, Heidelberg.

[69] Lucey P, Cohn J, Lucey S, Matthews I, Sridharan S, Prkachin KM. Automatically Detecting Pain Using Facial Actions. Int Conf Affect Comput Intell Interact Workshops. 2009 Dec 8;2009:1–8. doi: 10.1109/ACII.2009.5349321 ; PMCID: PMC3296481. 21278824 PMC3296481

[70] Rojo R, Prados-Frutos JC, López-Valverde A. Evaluación del dolor mediante el Sistema de Codificación de la Acción Facial. Revisión sistemática [Pain assessment using the Facial Action Coding System. A systematic review]. Med Clin (Barc). 2015 Oct 21;145(8):350–5. Spanish. doi: 10.1016/j.medcli.2014.08.010 Epub 2014 Nov 26. . 25433779

[71] Misiak B, Kotowicz K, Loska O, Stramecki F, Beszłej JA, Samochowiec J, et al. Decreased use of active coping styles contributes to elevated allostatic load index in first-episode psychosis. Psychoneuroendocrinology. 2018 Oct;96:166–172. doi: 10.1016/j.psyneuen.2018.06.021 Epub 2018 Jun 28. . 29980008

[72] McEwen BS, Stellar E. Stress and the individual. Mechanisms leading to disease. Arch Intern Med. 1993 Sep 27;153(18):2093–101. . 8379800

[73] Misiak B, Frydecka D, Zawadzki M, Krefft M, Kiejna A. Refining and integrating schizophrenia pathophysiology—relevance of the allostatic load concept. Neurosci Biobehav Rev. 2014 Sep;45:183–201. doi: 10.1016/j.neubiorev.2014.06.004 Epub 2014 Jun 17. . 24950476

[74] Juster RP, McEwen BS, Lupien SJ. Allostatic load biomarkers of chronic stress and impact on health and cognition. Neurosci Biobehav Rev. 2010 Sep;35(1):2–16. doi: 10.1016/j.neubiorev.2009.10.002 Epub 2009 Oct 12. . 19822172

[75] Nelson S, Bento S, Enlow MB. Biomarkers of Allostatic Load as Correlates of Impairment in Youth with Chronic Pain: An Initial Investigation. Children (Basel). 2021 Aug 18;8(8):709. doi: 10.3390/children8080709 ; PMCID: PMC8392178. 34438600 PMC8392178

[76] Berger M, Juster RP, Westphal S, Amminger GP, Bogerts B, Schiltz K, et al. Allostatic load is associated with psychotic symptoms and decreases with antipsychotic treatment in patients with schizophrenia and first-episode psychosis. Psychoneuroendocrinology. 2018 Apr;90:35–42. doi: 10.1016/j.psyneuen.2018.02.001 Epub 2018 Feb 5. . 29427955

[77] Chen E, Miller GE, Lachman ME, Gruenewald TL, Seeman TE. Protective factors for adults from low-childhood socioeconomic circumstances: the benefits of shift-and-persist for allostatic load. Psychosom Med. 2012 Feb-Mar;74(2):178–86. doi: 10.1097/PSY.0b013e31824206fd Epub 2012 Jan 27. Erratum in: Psychosom Med. 2015 Apr;77(3):344. Erratum in: Psychosom Med. 2015 Apr;77(3):344. ; PMCID: PMC3273596. 22286848 PMC3273596

[78] Keizer D, Fael D, Wierda JM, van Wijhe M. Quantitative sensory testing with Von Frey monofilaments in patients with allodynia: what are we quantifying? Clin J Pain. 2008 Jun;24(5):463–6. doi: 10.1097/AJP.0b013e3181673b80 . 18496312

[79] Riquelme I, Zamorano A, Montoya P. Reduction of pain sensitivity after somatosensory therapy in adults with cerebral palsy. Front Hum Neurosci. 2013 Jun 24;7:276. doi: 10.3389/fnhum.2013.00276 ; PMCID: PMC3690353. 23805086 PMC3690353

[80] Backonja MM, Attal N, Baron R, Bouhassira D, Drangholt M, Dyck PJ, et al. Value of quantitative sensory testing in neurological and pain disorders: NeuPSIG consensus. Pain. 2013 Sep;154(9):1807–1819. doi: 10.1016/j.pain.2013.05.047 Epub 2013 Jun 3. Erratum in: Pain. 2014 Jan;155(1):205. . 23742795

[81] Goodin BR, Quinn NB, King CD, Page GG, Haythornthwaite JA, Edwards RR, et al. Salivary cortisol and soluble tumor necrosis factor-α receptor II responses to multiple experimental modalities of acute pain. Psychophysiology. 2012 Jan;49(1):118–27. doi: 10.1111/j.1469-8986.2011.01280.x Epub 2011 Sep 6. ; PMCID: PMC3235230. 21895688 PMC3235230

[82] Palisano R., Rosenbaum P., Walter S., Russell D., Wood E., & Galuppi B. (1997). Gross motor function classification system for cerebral palsy. *Dev Med Child Neurol*, 39(4), 214–23. 9183258 10.1111/j.1469-8749.1997.tb07414.x

[83] Hidecker MJ, Paneth N, Rosenbaum PL, Kent RD, Lillie J, Eulenberg JB, et al. Developing and validating the Communication Function Classification System for individuals with cerebral palsy. Dev Med Child Neurol. 2011 Aug;53(8):704–10. doi: 10.1111/j.1469-8749.2011.03996.x Epub 2011 Jun 27. ; PMCID: PMC3130799. 21707596 PMC3130799

[84] Miró J, de la Vega R, Solé E, Racine M, Jensen MP, Gálan S, et al. Defining mild, moderate, and severe pain in young people with physical disabilities. Disabil Rehabil. 2017 Jun;39(11):1131–1135. doi: 10.1080/09638288.2016.1185469 Epub 2016 Jun 13. ; PMCID: PMC5553452. 27291566 PMC5553452

[85] de Knegt NC, Pieper MJ, Lobbezoo F, Schuengel C, Evenhuis HM, Passchier J, et al. Behavioral pain indicators in people with intellectual disabilities: a systematic review. J Pain. 2013 Sep;14(9):885–96. doi: 10.1016/j.jpain.2013.04.016 Epub 2013 Jul 3. . 23830762

[86] Choi JC, Chung MI, Lee YD. Modulation of pain sensation by stress-related testosterone and cortisol. Anaesthesia. 2012 Oct;67(10):1146–51. doi: 10.1111/j.1365-2044.2012.07267.x Epub 2012 Jul 16. Erratum in: Anaesthesia. 2013 May;68(5):550. . 22804789

[87] Durán-Carabali LE, Henao-Pacheco ML, González-Clavijo AM, Dueñas Z. Salivary alpha amylase and cortisol levels as stress biomarkers in children with cerebral palsy and their association with a physical therapy program. Res Dev Disabil. 2021 Jan;108:103807. doi: 10.1016/j.ridd.2020.103807 Epub 2020 Nov 6. . 33161308

[88] Theoharides TC, Tsilioni I, Bawazeer M. Mast Cells, Neuroinflammation and Pain in Fibromyalgia Syndrome. Front Cell Neurosci. 2019 Aug 2;13:353. doi: 10.3389/fncel.2019.00353 ; PMCID: PMC6687840. 31427928 PMC6687840

[89] Catley D, Kaell AT, Kirschbaum C, Stone AA. A naturalistic evaluation of cortisol secretion in persons with fibromyalgia and rheumatoid arthritis. Arthritis Care Res. 2000 Feb;13(1):51–61. doi: 10.1002/1529-0131(200002)13:1&lt;51::aid-art8&gt;3.0.co;2-q . 11094926

[90] McLean SA, Williams DA, Harris RE, Kop WJ, Groner KH, Ambrose K, et al. Momentary relationship between cortisol secretion and symptoms in patients with fibromyalgia. Arthritis Rheum. 2005 Nov;52(11):3660–9. doi: 10.1002/art.21372 . 16258904

