# Supplementary material for: Potential use of salivary TNF-α as a vaccine-induced pain biomarker in people with cerebral palsy and communication disorders
Source: PLoS One. 2024 Dec 27;19(12):e0308386. doi: 10.1371/journal.pone.0308386 (PMC11676923; doi:10.1371/journal.pone.0308386)
Supplement: S1 Checklist — (DOCX) [file pone.0308386.s001.docx]

STROBE Statement—checklist of items that should be included in reports of observational studies

|  | Item No | Recommendation |
| --- | --- | --- |
| **Title and abstract** | 1 | (*a*) Indicate the study’s design with a commonly used term in the title or the abstract  Page 1 & 2 |
|  |  | (*b*) Provide in the abstract an informative and balanced summary of what was done and what was found Page 2 |
| Introduction | | |
| Background/rationale | 2 | Explain the scientific background and rationale for the investigation being reported  Pages 3 |
| Objectives | 3 | State specific objectives, including any prespecified hypotheses Page 4 |
| Methods | | |
| Study design | 4 | Present key elements of study design early in the paper Page5 |
| Setting | 5 | Describe the setting, locations, and relevant dates, including periods of recruitment, exposure, follow-up, and data collection Page 5 |
| Participants | 6 | (*a*) *Cohort study*—Give the eligibility criteria, and the sources and methods of selection of participants. Describe methods of follow-up  *Case-control study*—Give the eligibility criteria, and the sources and methods of case ascertainment and control selection. Give the rationale for the choice of cases and controls Page 5  *Cross-sectional study*—Give the eligibility criteria, and the sources and methods of selection of participants |
|  |  | (*b*) *Cohort study*—For matched studies, give matching criteria and number of exposed and unexposed  *Case-control study*—For matched studies, give matching criteria and the number of controls per case |
| Variables | 7 | Clearly define all outcomes, exposures, predictors, potential confounders, and effect modifiers. Give diagnostic criteria, if applicable Page 5-9 |
| Data sources/ measurement | 8* | For each variable of interest, give sources of data and details of methods of assessment (measurement). Describe comparability of assessment methods if there is more than one group Page 5-9 |
| Bias | 9 | Describe any efforts to address potential sources of bias N.A |
| Study size | 10 | Explain how the study size was arrived at Page 5 |
| Quantitative variables | 11 | Explain how quantitative variables were handled in the analyses. If applicable, describe which groupings were chosen and why Page 10-11 |
| Statistical methods | 12 | (*a*) Describe all statistical methods, including those used to control for confounding Page 10-11 |
|  |  | (*b*) Describe any methods used to examine subgroups and interactions Page 10-11 |
|  |  | (*c*) Explain how missing data were addressed N.A |
|  |  | (*d*) *Cohort study*—If applicable, explain how loss to follow-up was addressed  *Case-control study*—If applicable, explain how matching of cases and controls was addressed Page 5  *Cross-sectional study*—If applicable, describe analytical methods taking account of sampling strategy |
|  |  | (*e*) Describe any sensitivity analyses |

Continued on next page

| Results | | |
| --- | --- | --- |
| Participants | 13* | (a) Report numbers of individuals at each stage of study—eg numbers potentially eligible, examined for eligibility, confirmed eligible, included in the study, completing follow-up, and analysed Page11 |
|  |  | (b) Give reasons for non-participation at each stage N.A |
|  |  | (c) Consider use of a flow diagram N.A |
| Descriptive data | 14* | (a) Give characteristics of study participants (eg demographic, clinical, social) and information on exposures and potential confounders Page 11 |
|  |  | (b) Indicate number of participants with missing data for each variable of interest N.A |
|  |  | (c) *Cohort study*—Summarise follow-up time (eg, average and total amount) |
| Outcome data | 15* | *Cohort study*—Report numbers of outcome events or summary measures over time |
|  |  | *Case-control study—*Report numbers in each exposure category, or summary measures of exposure Page 12-17 |
|  |  | *Cross-sectional study—*Report numbers of outcome events or summary measures |
| Main results | 16 | (*a*) Give unadjusted estimates and, if applicable, confounder-adjusted estimates and their precision (eg, 95% confidence interval). Make clear which confounders were adjusted for and why they were included Page11-17 |
|  |  | (*b*) Report category boundaries when continuous variables were categorized |
|  |  | (*c*) If relevant, consider translating estimates of relative risk into absolute risk for a meaningful time period |
| Other analyses | 17 | Report other analyses done—eg analyses of subgroups and interactions, and sensitivity analyses Page 17 |
| Discussion | | |
| Key results | 18 | Summarise key results with reference to study objectives Pages 17-19 |
| Limitations | 19 | Discuss limitations of the study, taking into account sources of potential bias or imprecision. Discuss both direction and magnitude of any potential bias Page 19 |
| Interpretation | 20 | Give a cautious overall interpretation of results considering objectives, limitations, multiplicity of analyses, results from similar studies, and other relevant evidence Page 17-19 |
| Generalisability | 21 | Discuss the generalisability (external validity) of the study results Page 20 |
| Other information | | |
| Funding | 22 | Give the source of funding and the role of the funders for the present study and, if applicable, for the original study on which the present article is based Page 20. |
