# Supplementary material for: Potential use of salivary TNF-α as a vaccine-induced pain biomarker in people with cerebral palsy and communication disorders
Source: PLoS One. 2024 Dec 27;19(12):e0308386. doi: 10.1371/journal.pone.0308386 (PMC11676923; doi:10.1371/journal.pone.0308386)
Supplement: S1 Table — (DOCX) [file pone.0308386.s002.docx]

***Table S1. Consort Flow chart of selection, enrollment, and adherence of participants in the study.*** *References: CP – Cerebral Palsy*

**Successfully assessed (*n*=30)**

**Follow up**

**Successfully assessed (*n*=30)**

**No randomization due the small size of the sample**

**No randomization due the small size of the sample**

**Randomization**

**Consent**

**Excluded (n=12) to match age and sex representation of individuals with Cerebral Palsy**

**Consented (*n*=42)**

**Screening**

**Excluded (*n*=5)**

- Recent surgery (n=1)
- Inflammatory process (n=4)

**Excluded (*n*=7)**

- Recent surgery (n=2)
- Inflammatory process (n=5)

**Consented (*n*=30)**

**Controls (*n*=115)**

**Individuals with Cerebral Palsy (*n*=75)**
